# Characterization and Cytotoxicity Studies of the Rare 21:4 *n*-7 Acid and Other Polyunsaturated Fatty Acids from the Marine Opisthobranch *Scaphander lignarius*, Isolated Using Bioassay Guided Fractionation 

**DOI:** 10.3390/md10122676

**Published:** 2012-11-26

**Authors:** Terje Vasskog, Jeanette H. Andersen, Espen Hansen, Johan Svenson

**Affiliations:** 1 Norut—Northern Research Institute, PO Box 6434, Tromsø Science Park, N-9294 Tromsø, Norway; Email: Terje.vasskog@norut.no; 2 Department of Pharmacy, University of Tromsø, Breivika, N-9037 Tromsø, Norway; 3 Marbio, University of Tromsø, Breivika, N-9037 Tromsø, Norway; Email: Jeanette.h.andersen@uit.no (J.H.A.); Espen.hansen@uit.no (E.H.); 4 Department of Chemistry, University of Tromsø, Breivika, N-9037 Tromsø, Norway

**Keywords:** opisthobranch, cytotoxic, PUFA, *Scaphander lignarius*, ω7 fatty acid

## Abstract

The marine opisthobranch *Scaphander lignarius* has been analyzed in the systematic search for novel bioactive compounds in Arctic marine organisms using bioassay guided fractionation. A number of highly cytotoxic fractions were shown to contain mainly polyunsaturated fatty acids (PUFAs). Selected PUFAs were isolated and identified using both liquid chromatography-mass spectrometry (LC-MS) and nuclear magnetic resonance (NMR). It was shown that the opisthobranch contained unusual PUFAs such as several ω3 fatty acids and the ω7 heneicosa-5,8,11,14-tetraenoic acid (21:4 *n*-7) not isolated before. The organism was shown to be a very rich source of PUFAs and the activity of the isolated compounds against a range of human cancer cell lines (melanoma, colon carcinoma and breast carcinoma) is further reported. The ω7 PUFA was significantly more cytotoxic in comparison with reference ω6 arachidonic and ω3 eicosapentaenoic acid. A noteworthy non-selective cytotoxicity against normal lung fibroblasts was also established. The paper contains isolation protocols in addition to cytotoxicity data of the isolated compounds. The potential of marine mollusks as a source for rare PUFAs is also discussed.

## 1. Introduction

The marine environment is highly competitive and being able to produce compounds providing an adaptive advantage is highly beneficial for any marine organism. Such compounds are fundamentally interesting to study but they also represent entities with significant potential for several industrial and medical applications as they have been chemically optimized during millions of years of evolution and selection [1]. Marine mollusks represent a particularly promising group of organisms in the search for new bioactive compounds. In fact, the majority (35%) of the 20 marine natural products currently approved as drugs or in clinical trials have their origin in collected mollusks [2,3]. Most marine drugs are used in the treatment of different types of cancer although other disease states such as schizophrenia, Alzheimer’s and hypertriglyceridemia are also represented. 

**Figure 1:**
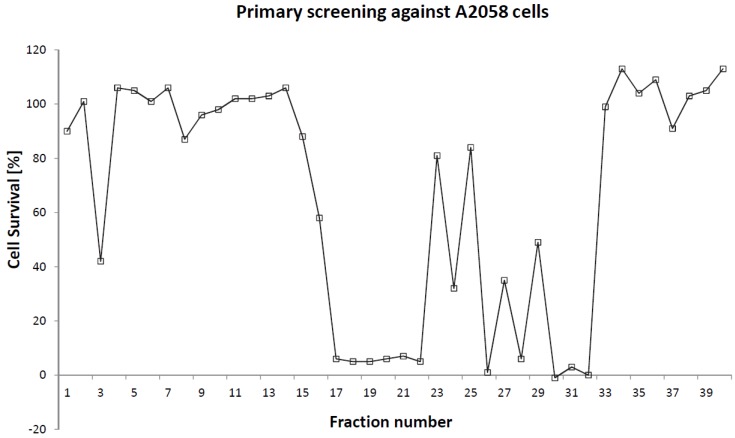
Results from the primary screening of the *Scaphander lignarius* acetonitrile extract against human A2058 melanoma cells. The compounds in the current study were isolated from fractions 26–32.

The marine organisms inhabiting the long and twisting coastline surrounding the northern parts of Norway are currently being investigated in a large scale search for novel compounds for drug discovery and development [4]. Numerous mollusks have been gathered and analyzed in the course of the methodical collection and screening of Arctic marine organisms. Bioassay guided fractionation is employed to allow isolation of compounds displaying promising bioactivities. Recently, an organic extract of the opisthobranch *Scaphander lignarius* generated fractions with high cytotoxic activities (Figure 1). 

**Figure 2:**
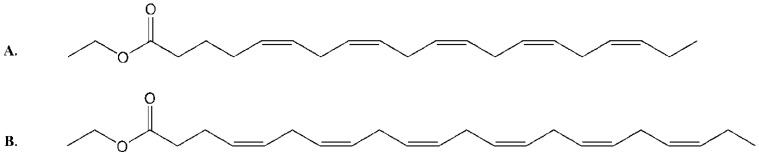
Chemical structures of the fatty acid ethyl esters of: (**A**) eicosapentaenoic acid (EPA) and (**B**) docosahexaenoic acid (DHA), the main active compounds in Lovaza^®^/Omacor^®^.

The active components in the fractions were shown to be a group of PUFAs, present in high concentrations. Marine mollusks and invertebrates are rich in lipids and they have been extensively studied over the years and comprehensively reviewed by Bergé and Barnathan [5]. Numerous bioactive fatty acids have been shown to display both antimicrobial [6] and cytotoxic bioactivities in addition to their perhaps more established ability to alleviate the burden and lower the risk of cardiac failure and obesity [7,8,9,10]. In addition to displaying cytotoxic activities on their own, several PUFAs have also been reported to synergistically potentiate the efficacy of established anticancer drugs through several mechanisms [11,12,13]. One of the seven food and drug administration (FDA) approved drugs of marine origin today is a mixture of ω3-acid ethyl esters (Lovaza^®^/Omacor^®^) displayed in Figure 2, used to treat hypertriglyceridemia and cardiovascular disease and illustrates the pharmaceutical potential of fatty acids of marine origin.

Any biological source able to generate large amounts of unusual, biologically active fatty acids is important to further study as their biological properties are poorly known [14]. In the current investigation, several common PUFAs and an unusual ω7 PUFA (not previously isolated) were identified in a *Scaphander lignarius* extract. Three representative PUFAs, arachidonic acid (20:4 *n*-6) (ARA), eicosapentaenoic acid (20:5 *n*-3) (EPA) and the rare heneicosa-5,8,11,14-tetraenoic acid (21:4 *n*-7) (HTA) were isolated and selected for dose response cytotoxicity studies against a set of human cancer cell lines and structure activity relationship (SAR) establishment. The current paper describes the isolation and cytotoxic bioactivities of these selected PUFAs against several human cancer cell lines. It represents the first study of the fatty acid composition of *Scaphander lignarius* and the first assessment of the cytotoxic properties of an oddly numbered ω7 PUFA*.*

## 2. Results and Discussion

**Figure 3:**
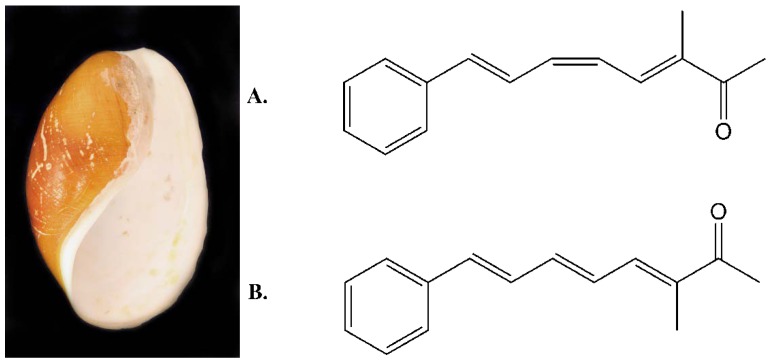
The opisthobranch *Scaphander lignarius* and the chemical structures of the polyketides: (**A**) lignarenone A and (**B**) lignarenone B previously isolated from it.

The opisthobranches represent a large group of marine mollusks belonging to the gastropods with wide geographic distribution and diverse appearances [15]. The opisthobranch *Scaphander lignarius* in the current study has previously been described as an abundant Mediterranean mollusk [16] but it clearly has a much wider geographical distribution as the biological material used in the current study was collected some 70 degrees north in Arctic waters. It has previously been show to contain a family of conjugated polyketides known as lignarenones, shown in Figure 3, suggested by Cimino to function as alarm pheromones [17,18].

### 2.1. Isolation and Structural Determination

**Figure 4:**
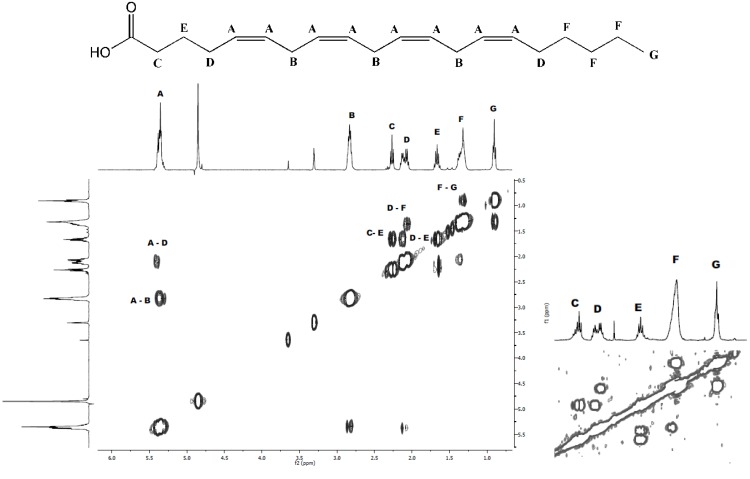
2D COSY spectrum and the atomic labeling of the characteristic PUFA proton signals (arachidonic acid (20:4 *n*-6) (ARA)) illustrating the correlations between the “**F**” protons and the terminal “**G**” methyl group and the “**D**” protons. Insert from heneicosa-5,8,11,14-tetraenoic acid (21:4 *n*-7) (HTA) COSY illustrates the differences in “**F**” intensity allowing for accurate placement of the additional methylene in HTA.

**Figure 5:**
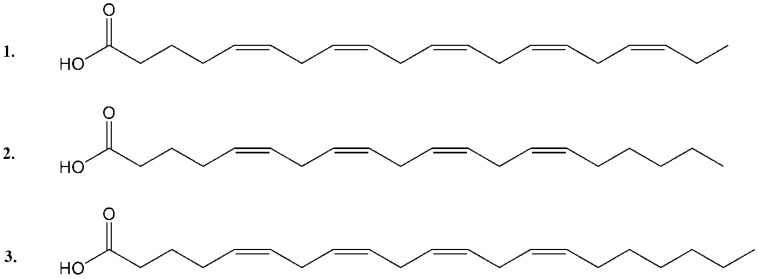
Structure of the isolated PUFAs: (**1**) EPA; (**2**) ARA and (**3**) HTA.

Based on what has previously been reported about the content of *Scaphander lignarius* it was anticipated that potentially new lignarenone derivatives could be accountable for the cytotoxic activity in the extract as polyketides have been shown to display a wide range of biological activities [19,20]. Three of the major constituents (**1**–**3**) found in the active fractions (fractions 26–32 in Figure 1) were isolated from the organic extract. MS-Data was not solely used to unambiguously identify the compounds although the accurate masses of the major compounds could be tentatively assigned to PUFAs. The final identity of the bioactive compounds was established employing NMR analysis. While several ^1^H signals at a glance could correlate to a lignarenone-like structure this was ruled out during closer inspection of the ^1^H NMR data which displayed the “classic” set of PUFA signals (Figure 4). **1** and **2** were rapidly assigned to ARA and EPA ([M − H]^−^ 303.2339, calculated 303.2324 and 301.2186, calculated 301.2168, respectively). The NMR spectra of **1** and **2** could be used to establish the location of the extra methylene in **3** ([M − H]^−^ 317.2497, calculated 317.2481) using ^1^H and two-dimensional correlation spectroscopy (COSY) experiments by observing differences in the number of “**F**” methylene protons displayed below in Figure 4 and **3** was subsequently identified as HTA. The NMR spectra of ARA and HTA were, apart from the difference in number of “**F**” methylene protons, identical. The structure of compounds **1**–**3** are presented in Figure 5.

The fatty acid profile of the extract was further established once the isolation and structural elucidation revealed PUFAs as the source of the cytotoxic bioactivity and is compiled in Table 1. 

**Table 1:** Major fatty acid constituents in *Scaphander lignarius*.

Fatty acid	Monoisotopic mass	Relative area	%
			
Monoenonic			
14:1	226.2016	137	0.03
16:1	254.2328	1587	0.31
18:1	282.2640	1026	0.20
20:1	310.2952	1182	0.23
			
NMID ^a^			
18:2	280.2484	311	0.06
20:2	308.2796	642	0.13
22:2	336.3108	210	0.04
			
Polyenonic			
16:3	250.2016	883	0.17
18:3	278.2328	787	0.16
20:3	306.2640	660	0.13
18:4	276.2172	1970	0.39
20:4 *n*-6	304.2484	88921	17.53
21:4 *n*-7	318.2640	46741	9.22
22:4 (2 isomers)	332.2796	6986 + 8824	3.12
20:5 *n*-3	302.2328	201409	39.72
22:5 (3 isomers)	330.2640	31588 + 4406 + 4295	7.95
22:6	328.2484	104560	20.61
			
Sum		507123	100.00
			

^a^ Non-Methylene-Interrupted dienoic fatty acids.

**Figure 6:**
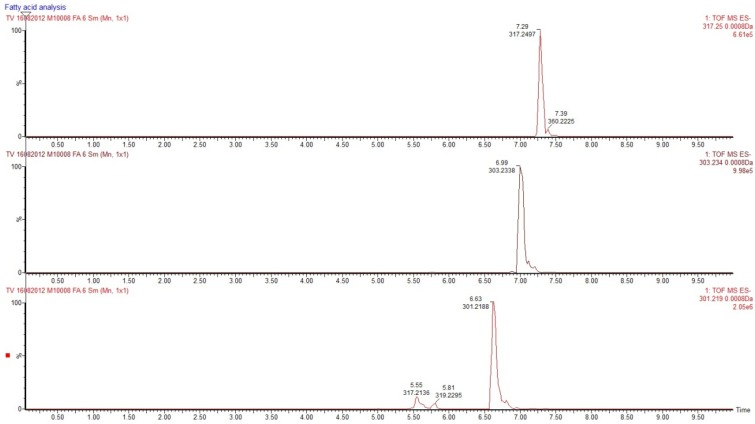
Ion chromatograms of the three isolated PUFAs: Top, HTA (*m/z* 317.2497), middle, ARA (*m/z* 303.2338) and bottom EPA (*m/z* 301.2188).

The fatty acid distribution profile of the *Scaphander lignarius* extract corresponds well with that of other marine mollusks [21,22]. The majority of the fatty acids are PUFAs with ARA, EPA and DHA being the most prevalent accounting for nearly 80% of the fatty acid content. A low relative content of the less common NMIDs has been reported in other marine mollusks even though they can be a rich source [22,23]. Of particular interest is the high content of HTA (9.22%) which has mainly only been reported in minute amounts before and not been isolated. It is clear that this rare ω7 fatty acid play an important role in *Scaphander lignarius*. Unexpectedly, no saturated fatty acids were detected which may be a reflection of the methods used to extract the organism. The semi-automated high-throughput bioassay guided fractionation strategy employed is focused on isolating drug-like compounds of certain molecular mass and polarity [4]. Such a strategy is necessary in order to analyze the vast number of organisms collected, clearly at the potential expense of overlooking interesting compounds not fulfilling the molecular requirements for isolation. These challenges associated with high-throughput isolation of natural products are established [24]. No attempts were thus made at isolating additional fatty acids potentially discarded during the extraction steps and the fatty acid distribution presented in Table 1 is only representative for the aqueous acetonitrile fraction as described in the isolation section. Ion chromatograms of the isolated PUFAs are presented in Figure 6.

### 2.2. Cytotoxicity

It is well-established that a regular intake of PUFA rich food can lower the risk of obesity, cardiac failure and certain cancers types [5,25,26]. The association between a high intake of ω3 PUFAs and a reduction of major cardiovascular disease events is nevertheless currently under significant debate [27]. In addition, both saturated fatty acids and PUFAs have been intensively studied as anticancer drugs [28,29]. Several modes of action are suggested in the literature with increased lipid peroxidation, modulation of the cyclooxygenase-2/prostaglandin E2 (COX/PGE_2_) pathway and inhibition of topoisomerases, crucial for topological DNA processing, being well-established cellular targets [29,30]. A certain group of fatty acids, the non-methylene-interrupted fatty acids (NMI-FA) are particularly common in marine organisms and they have recently been shown to display interesting biological activities [23,31]. These compounds were however only present in minute amounts in the *Scaphander lignarius* extract which prevented isolation and accurate analysis of double bond placement.

Due to a lowered enzymatic ∆6- and ∆5-desaturase activity, certain tumor types are deficient in PUFAs and this compositional difference can allow cytotoxic PUFAs to be used as selective cytotoxic agents [32]. Yoshida hepatoma cells does for example only contain half the amount of ARA in comparison with normal hepatocytes [30]. Exposure to elevated concentrations of ARA, EPA and γ-linolenic acid have all been shown to induce tumoral apoptosis by increasing the lipid peroxidation and subsequent generation of free radicals such as the highly reactive superoxide anion and hydrogen peroxide [33,34,35].

Tissue selectivity is of major concern for any cytotoxic agent and several studies have shown that PUFAs indeed are toxic to “normal” cells, in addition to tumor cells at sufficiently high concentrations. Das have reported toxicity values (50% dead cells after 7 days incubation) for EPA and ARA against normal human fibroblasts (CCD-41-SK) at ~60 µg/mL and ~30 µg/mL respectively. Those concentrations were some 2–3 times higher than the concentration needed to kill 100% of human breast tumor cells (ZR-75-1) [36]. Stonik *et al.* further reported significant hemolytic activity at concentrations as low as 1.5 µg/mL for the rare (5*Z*,9*Z*)-22-methyl-5,9-tetracosadienoic acid isolated from the marine sponge *Geodinella robusta *[37]. Nevertheless, most studies suggest an increased sensitivity to PUFAs for tumor cells in comparison with normal cells. In addition, a number of novel tumor delivery strategies, such as intratumoral injections, PUFA/growth factor conjugation and PUFA/cancer drug complexes are under development to allow selective tumoral delivery [30,38] of PUFAs.

**Figure 7:**
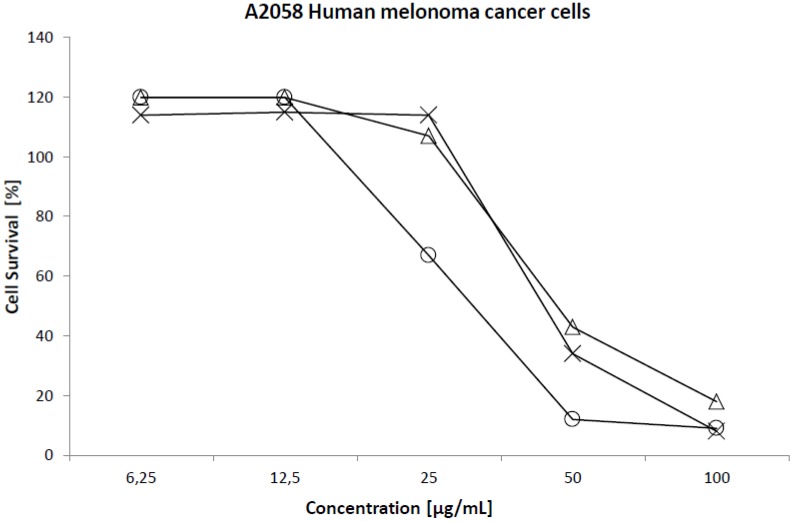
Illustration of the dose dependent PUFA induced killing of A2058 melanoma cells by EPA (-∆-), ARA (-x-) and HTA (-o-).

Three of the major constituents in the *Scaphander lignarius* extract, ARA, EPA and HTA were chosen for further cytotoxicity studies against a set of cancer and normal cell lines as described in the methods section. By including an ω3, an ω6 and an ω7 PUFA in the cytotoxicity studies, it is conceivable to establish the potential structure activity relationship (SAR). Despite its abundance, DHA was not isolated as EPA was already included as representative ω3 PUFA. ARA and EPA are well studied PUFAs while cytotoxicity studies of the rare ω7 HTA has not been described in the literature before [5,28]. All the compounds were able to kill cancer cells in a dose dependent manner exemplified below in Figure 7 for A2058 cells. From the dose-response curves it was possible to calculate the IC_50_ values for the individual PUFAs and those figures are presented in Table 2.

**Table 2:** Cytotoxicity of ARA, EPA and HTA against selected cell lines.

Cell line	50% Cytotoxicity (µg/mL)		
	EPA	ARA	HTA
Normal lung fibroblast (MRC5)	90	90	42
Colon carcinoma (HT29)	93	76	41
Melanoma (A2058)	45	43	31
Breast carcinoma (MCF7)	66	68	44

All the studied PUFAs were shown to be toxic to both tumor and normal cells. The IC_50_ values were slightly higher than those reported for EPA and ARA by Das against normal fibroblasts and breast tumors [36]. Those studies were however performed with significantly longer cellular exposure times in comparison with the current investigation. The IC_50_ figures indicate that HTA is nearly twice as cytotoxic in comparison to EPA and ARA. A preference for tumoral cells over normal cells was seen for both EPA and ARA. This cellular selectivity was most pronounced for the melanoma (A2058) and breast carcinoma (MCF7) cells. A lower content of super oxide dismutase in certain cancer cells has been suggested as an explanation of the differences in cellular sensitivity to PUFAs [36]. HTA displayed no obvious cellular selectivity.

The structural differences between the three investigated PUFAs are minor and lie mainly in the number and placement of the double bonds. HTA with its extra methylene contains an additional carbon while EPA contains five double bonds. These differences are reflected in their hydrophobicity and theoretical logP coefficients which range from 5.85, 6.16 to 6.58 for EPA, ARA and HTA, respectively. The calculated logP values (calculated using Chemdraw ultra 10.0) correlate well with their experimental C-18 retention times: 6.63, 6.99 and 7.29 min respectively for EPA, ARA and HTA. The uptake and distribution of fatty acids differ between normal cells and tumor cells and it has previously been shown to influence the extent of cellular death. The increased hydrophobicity appears to provide HTA with augmented cytotoxicity. The lack of distinction between normal cells and tumor cells further implies that the mode of action may be dictated by a feature or mechanism that is mutual in both normal and tumoral cells. A plausible common target could be the cellular membrane. Being an exogenic PUFA, its membrane insertion is likely to yield membrane disruption once a threshold concentration has been reached. Established intracellular events such as increased lipid peroxidation and superoxide radical generation are also possible [29]. The higher hydrophobicity may further allow an increased cellular uptake of HTA compared to EPA and ARA which could be an additional contribution to the apparent higher cytotoxicity. Insufficient amounts of HTA prevented mechanistic investigations such as co-incubation with antioxidants and calcein leakage studies.

HTA has been identified in minute amounts by MS in extracts of cold-seep mussels and in the deep sea-vent crab *Shinkaia crosnieri*, by Saito [22,39] and in an extract from the sponge *Hymeniacidon sanguinea *[40] but it has never been isolated and biologically tested. Very recently, HTA was reported to be a main constituent (7.5% of total fatty acids) of an Australian thraustochytrid (*Thraustochytrium* sp.) from south east Tasmania [41]. The fatty acid composition of 36 different thraustochytrid strains was evaluated as sources for the production of biodiesel and 12 of those where shown to contain HTA. Of those 12, only one contained HTA concentrations comparable to *Scaphander lignarius*. Being an oddly numbered ω7 PUFA it represents an interesting target for studies as it may yield additional insight into the role and potential of PUFAs as cytotoxic agents. Oddly numbered fatty acids are generally rare in Nature and hence less studied as cytotoxic agents both due to the low abundance but also on the inherent difficulties associated with their detection and identification [14,42]. 

PUFAs are produced via several biosynthetic pathways depending on substrate availability [41,43]. In addition to the conventional elongation and desaturation pathway they may also utilize the polyketide synthase pathway. Oddly numbered PUFAs have been isolated from crabs, crayfish and bivalves and it has been suggested that they have been accumulated in these organisms from their diet [14]. Oddly numbered PUFAs have for this reason been heralded as useful biomarkers for marine food web studies [43]. That is a likely scenario also for *Scaphander lignarius* and the high HTA content may be a reflection of a local diet rich in HTA producing microorganisms. The data presented in the current study also indicate that they may be useful cytotoxic agents given their increased cytotoxic activity. Marine mollusks appear to be the richest source so far encountered and further studies will reveal if these compounds are endogenous or a result of their diet and local surrounding conditions.

During the course of the screening process, the PUFA extract was also screened against a set of bacterial strains composed of *Escherichia coli*, *Staphylococcus aureus*, Methicillin resistant *Staphylococcus aureus*, *Pseudomonas aeruginosa*, and *Enterococcus faecalis*. No apparent bioactivity against any of the tested strains was seen (data not shown). 

## 3. Experimental Section

### 3.1. Collection

The biological material was collected by Agassiz dredging at a depth of 204 m in the Porsangerfjord (70°48′N, 26°10′E) in February 2008. The samples were taxonomically identified on board and stored at −23 °C until further used. Voucher specimen deposited at the national biobank Marbank, Norway.

### 3.2. Extraction and Fractionation

249.5 g of freeze-dried sample of *Scaphander lignarius* (organism wet weight: 1081.5 g) was ground and extracted twice with ultra-pure water (Milli-Q, Millipore) (24 h and 30 min) at 5 °C in the dark. After centrifugation and supernatant removal, the remaining sediment was freeze-dried, ground, and extracted twice with a 1:1 (v:v) mixture of dichloromethane and methanol (MeOH) (24 h and 30 min) at 5 °C in the dark. The mixture was further vacuum filtered through a Whatman (Ø 125 mm nr. 3) filter (Little Chalfont, UK) and the resulting supernatant was reduced to a concentrated organic extract (27.0 g) under vacuum. 300 mg of the extract was dissolved in 3 mL of hexane and extracted twice with a mixture of 90% acetonitrile and 10% purified water. The acetonitrile phases were collected and reduced to a concentrated liquid which was diluted with 50% aqueous acetonitrile (v:v) and fractionated into 40 fractions on HPLC using a Waters XTerra C18 column (10 µm particle size, 10 × 300 mm) employing a linear gradient from 20% to 100% acetonitrile in water (both containing 0.05% formic acid) over 30 min, and then holding at 100% acetonitrile for 10 min. The flow was set to 6 mL/min, and 40 one-minute fractions were collected. The HPLC-system consisted of a 2767 solvent manager, a 2996 photodiode array UV-detector and 600E pump, and was controlled by MassLynx 4.1 software and the FractionLynx application manager (Waters). The fractions were screened against A2058 cells according to the methodology described in paragraph 3.5 below and shown in Figure 1.

### 3.3. Isolation

10 g of the organic extract was used for the isolation of the fatty acids which was performed on a Waters (Milford, MA, USA) 2695 separation module employing two different gradients and columns. The initial separation was performed on a Waters Xbridge BEH C-18 OBD preparative column (5 μm particle size, 19 × 250 mm). The mobile phases consisted of A: 0.02% NH_3_ in purified water, and B: 90% acetonitrile, 10% purified water (containing 0.02% NH_3_). The fatty acids were collected with a Waters fraction collector II after detection with a Waters 2998 photo diode array detector. Mass spectrometric analysis of the fractions revealed that the fractions contained impurities, and a second clean-up step was necessary. The second preparative HPLC run was performed on a Waters Xbridge BEH C-18 OBD preparative column (5 μm particle size, 10 × 250 mm,). The mobile phases consisted of A: 0.1% trifluoroacetic acid (TFA) in purified water, and B: 90% acetonitrile, 10% purified water and 0.1% TFA. The gradients used for isolation are presented in Table 3. Amount PUFA isolated: EPA, 9.5 mg, ARA, 7.5 mg and HTA, 1.5 mg.

**Table 3:** Chromatography gradients employed for FA isolation.

	Time (min)	Flow (mL/min)	A (%)	B (%)
				
Gradient 1 ^a^	0	3	90	10
	0.5	10	90	10
	30	10	40	60
	35	10	5	95
	45	10	5	95
				
Gradient 2 ^b^	0	3	50	50
	0.5	7	50	50
	30	7	10	90
	40	7	10	90
				

^a^ A: 0.02% NH_3_ in purified water, and B: 90% acetonitrile, 10% purified water (containing 0.02% NH_3_); ^b^ A: 0.1% TFA in purified water, and B: 90% acetonitrile, 10% purified water and 0.1% TFA.

### 3.4. Structure Determination

The structures of the isolated PUFAs were confirmed using ultra-performance liquid chromatography (UPLC)-HR-MS analysis and nuclear magnetic resonance (NMR) spectroscopy. UPLC-HR-MS analysis was performed on a Waters Acquity I-class UPLC and a Waters Xevo G2 Q-ToF mass spectrometer (see further details below). The data was processed using MassLynx software version 4.1. NMR spectra were obtained on samples dissolved in MeOH-*d*_4_ (Sigma) on a Varian 400 MHz Spectrometer (Palo Alto, USA). One-Dimensional ^1^H and ^13^C NMR experiments, and two-dimensional ^1^H-^1^H COSY experiments were performed to verify the structures. MestreNova 5.2.4 software was used to process the data. **EPA**: ^1^H NMR (400 MHz, cd_3_od) *δ* 5.35 (m, 10H), 2.84 (m, 8H), 2.29 (t, *J* = 7.5 Hz, 2H), 2.11 (m, 4H), 1.66 (p, *J* = 7.4 Hz, 2H), 0.90 (t, *J* = 6.8 Hz, 3H). **ARA**: ^1^H NMR (400 MHz, cd_3_od) *δ* 5.36 (m, 8H), 2.83 (m, 6H), 2.30 (t, *J* = 7.5 Hz, 2H), 2.14 (q, *J* = 7.4 Hz, 2H), 2.07 (q, *J* = 7.4 Hz, 2H), 1.67 (p, *J* = 7.4 Hz, 2H), 1.32 (m, 6H), 0.90 (m, 3H). **HTA**: ^1^H NMR (400 MHz, cd_3_od) *δ* 5.36 (m, 8H), 2.83 (m, 6H), 2.27 (t, *J* = 7.5 Hz, 2H), 2.13 (q, *J* = 7.4 Hz, 2H), 2.07 (q, *J* = 7.4 Hz, 2H), 1.67 (p, *J* = 7.4 Hz, 2H), 1.35 (m, 8H), 0.90 (t, *J* = 6.8 Hz, 3H).

The fatty acid distribution profile was determined by UPLC-MS on a Waters Xevo G2 Q-ToF with negative electrospray ionization. The separation was performed on a Waters Acquity UPLC H-class, fitted with a Waters Acquity UPLC BEH C18 column (1.7 µm particle size, 2.1 × 100 mm) Mobile phase A consisted of H_2_O + 0.1% formic acid, and mobile phase B of acetonitrile + 0.1% formic acid. A gradient elution was employed, starting at 5% B, with a linear increase to 95% B at 7 min. 95% B was maintained for 3 min and the total runtime was 10 min. A similar ionization efficiency of all the fatty acids is a prerequisite for the given distribution profile. Identification of the fatty acids not isolated was based on retention times, elemental composition calculated by the MassLynx 4.1 software and the characteristic neutral losses of 18, 44, 78 and 98 during MS/MS fragmentation which confirmed the presence of the respective fatty acids.

### 3.5. Cytotoxicity Assays

The effect of the PUFAs on cell viability was tested against three cancer cell lines: Human melanoma (A2058, ATCC: CRL-1145™), human breast carcinoma (MCF7, ATCC: HTB-22™), and human colon carcinoma (HT29, ATCC: HTB-38™). In addition, non-malignant lung fibroblasts (MRC5, ATCC: CCL-171™) were used as toxicity control. Cell lines, suspended in RPMI-1640 or E-MEM medium (Biochrom AG) with 10% fetal bovine serum medium and 10 µg/mL gentamicin, were seeded in 96-well microtitre plates at 2,000 cells/well. The cell lines were incubated for 24 h before PUFAs (concentration range 6.25–100 µg/mL) were added and then incubated for 72 h at 37 °C in a humidified atmosphere of 5% CO_2_. The PUFAs were added to the cells in triplicate. Cell viability was determined by a colorimetric [3-(4,5-dimethylthiazol-2-yl)-5-(3-carboxymethoxyphenyl)-2-(4-sulfophenyl)-2*H*-tetrazolium] (MTS) assay. At the end of the exposure time, 10 µL Cell Titer 96^®^ Aqueous One Solution Reagent (Promega, USA) was added to each well, and the plates were incubated for 1 h before absorbance was measured using a DTX multimode detector (Beckman Coulter, CA, USA) at 485 nm. Cells in RPMI-1640 medium were used as negative control, and cells treated with Triton^®^ X-100 (Sigma-Aldrich) reagent was used as positive control. Relative cellular survival was determined by using the measured optical density (OD) and was calculated as follows: Cell survival (%) = (OD treated well − OD positive control)/(OD negative control − OD positive control) × 100.

## 4. Conclusions

The fatty acid composition in an extract of *Scaphander lignarius* collected in Arctic waters has been investigated. The mollusk was rich in ARA, EPA, DHA and the unusual ω7 HTA. Selected PUFAs were isolated and their cytotoxic activity was evaluated against a range of adherent human cancer cell lines. HTA was shown to be a significantly more potent cytotoxic agent than ARA and EPA. The cytotoxicity was not selective and normal fibroblasts were equally sensitive to the increased cytotoxicity of HTA. Tumor selective delivery systems would have to be employed to benefit from the cytotoxic properties of HTA without collateral damage to surrounding tissue. This is the first study of the cytotoxic action of an ω7 PUFA isolated from a particularly rich marine source.
